# C to U RNA editing of MFN1 is regulated by ADARB1 and associates with favourable prognosis in chronic lymphocytic leukemia

**DOI:** 10.1038/s41598-025-15666-6

**Published:** 2025-08-14

**Authors:** Alejandra Gonzalez Martinez, Franz Josef Gassner, Dominik Baumgartner, Philine Hoven, Ekaterina Akimova, Markus Steiner, Stephanie Schlichtner, Stefanie Rauscher, Ferran Nadeu, Elias Campo, Richard Greil, Alexander Egle, Lisa Pleyer, Nadja Zaborsky, Roland Geisberger

**Affiliations:** 1 Department of Internal Medicine III With Haematology, Medical Oncology, Haemostaseology, Infectiology and Rheumatology, Oncologic Center; Paracelsus Medical University, Salzburg Cancer Research Institute - Laboratory for Immunological and Molecular Cancer Research (SCRI-LIMCR), Cancer Cluster Salzburg, 5020 Salzburg, Austria; 2 Department of Bioscience, Paris-Lodron University Salzburg, 5020 Salzburg, Austria; 3 Molecular Pathology of Lymphoid Neoplasms Program, Institut d’Investigacions Biomèdiques August Pi I Sunyer (IDIBAPS), Barcelona, Spain; 4 Centro de Investigación Biomédica en Red de Cáncer (CIBERONC), Madrid, Spain; 5 Hospital Clínic of Barcelona, Barcelona, Spain; 6 Universitat de Barcelona, Barcelona, Spain

**Keywords:** RNA editing, ADARB1, APOBEC, CLL, RNA, Chronic lymphocytic leukaemia, RNA editing

## Abstract

Cytidine to uridine (C-to-U) as well as adenosine to inosine (A-to-I) RNA editing denotes the posttranscriptional modification of RNA by specialized RNA deaminases. As RNA editing alters the sequence of the RNA, it can affect splicing, stability, miRNA binding and may also lead to recoding of the translated protein. Recently, we analysed recoding A-to-I RNA editing in chronic lymphocytic leukaemia (CLL) and could define prognostically relevant editing patterns. However, disease relevant C-to-U RNA editing in CLL remained unexplored. In this study, we examined C-to-U RNA editing in CLL and discovered a recoding RNA editing site within the *MFN1* gene (hg38; chr3:179,375,230), which has recently been described as RNA editing site in brain samples. We found that *MFN1* editing was not only present in CLL samples but also in naive B cell subsets, primarily occurred at unspliced RNA and correlated with intron retention. We further identified catalytically active ADARB1 as an essential regulator for *MFN1* editing. Finally, *MFN1* editing correlated with prolonged time to treatment and overall survival in CLL patients. Summarizing, we identified a novel ADARB1 function as C to U editing regulator, which regulates *MFN1* splicing and MFN1 S329L recoding with pathogenic relevance in CLL.

## Introduction

Generally, RNA editing comprises posttranscriptional modifications of single bases within RNA molecules. In mRNA, the most prominent RNA editing events are deamination of either adenosine or cytosine, catalyzed by ADARs (adenosine deaminases that act on RNA; ADAR1 and ADAR2) and by AID/APOBEC (activation-induced deaminase/Apolipoprotein B mRNA editing enzyme, catalytic polypeptide-like) family members. While deamination of adenosine yields an inosine (decoded as guanosine), deamination of cytosine leads to uracil, whereupon ADAR dependent RNA editing is referred to as A > I (or A > G) editing and AID/APOBEC dependent editing as C > U editing. While A > I editing is the most common type of RNA editing in metazoan, AID/APOBEC dependent C > U editing is restricted to mammals^1^. Recent advances in massive parallel sequencing allowed systematic detection of single base differences from matched DNA/RNA samples, leading to description of an increasing number of individual RNA editing sites in the human genome^2^. Although most of these editing sites are confined to non-coding transcripts and are important to deactivate endogenous retroviral elements and transposons, many RNA editing sites were discerned in mRNAs, changing the coding sequence itself or affecting miRNA binding sites or splicing patterns^1,3^.

In humans, the AID/APOBECs comprise 11 family members, AID, APOBEC1 (A1), A2, A3A, A3B, A3C, A3DE, A3F, A3G, A3H and A4, with all of them (except for A4) showing C-to-U deaminase activity on single-stranded DNA or RNA^4,5^. So far, RNA editing activity has been reported for A1, A2, A3A and A3G. Originally, A1 was found to edit the RNA of ApoB (apolipoprotein B) in the small intestine, leading to a premature stop codon and expression of a truncated APOB protein, important for lipid metabolism^6^. Since then, A1 was found to have hundreds of targets in RNAs from immune cells and neurons, important for inflammation and brain function^7,8^. While A2 was originally proposed to have a role in muscle development without evident roles in DNA/RNA editing, A2 transgenic mice showed A2 dependent RNA editing of specific transcripts and contributed to tumorigenesis^5,9^. Furthermore, A3A was shown to edit hundreds of gene transcripts during macrophage polarization and overexpression of A3A and A3G in cell lines induced C > U editing in thousands of target genes^10–13^.

It has been noticed that RNA editing is frequently dysregulated in cancer and may critically contribute to cancer pathogenesis or serve as prognostic indicator^14,15^. Recently, we defined a catalogue of A-to-I editing events in chronic lymphocytic leukemia (CLL), a B cell malignancy of the elderly with high incidence. There, we could characterize CLL-specific RNA and miRNA editing and extracted RNA editing patterns with prognostic impact^16,17^. In the present study, we analysed C-to-U editing in CLL and discovered a C-to-U editing site within the *MFN1* gene, which leads to S329L recoding of MFN1 (mitofusin-1), a protein critically involved in mitochondria network formation^18^. This editing site has been recently detected in brain samples in context of schizophrenia and was shown to affect MFN1 function in mitochondrial fusion^19^. Mitochondrial dysfunction has been described in many diseases including cancer^20^. As mitochondrial networks are implicated in many biological processes including cell death, proliferation, migration and calcium signaling^21^, *MFN1* editing can influence these pathways thereby affecting cellular homeostasis. Here, we show that *MFN1* is highly edited in naïve B cells and in a subset of CLL cell samples. We found that *MFN1* editing is associated with retention of the intron upstream of the editing site and we identified the A > I editing enzyme ADARB1 as a regulator for *MFN1* C-to-U editing. Finally, we found that *MFN1* editing is associated with favourable prognosis in CLL.

## Results

### C-to-U editing is observed in CLL

**Fig. 1:**
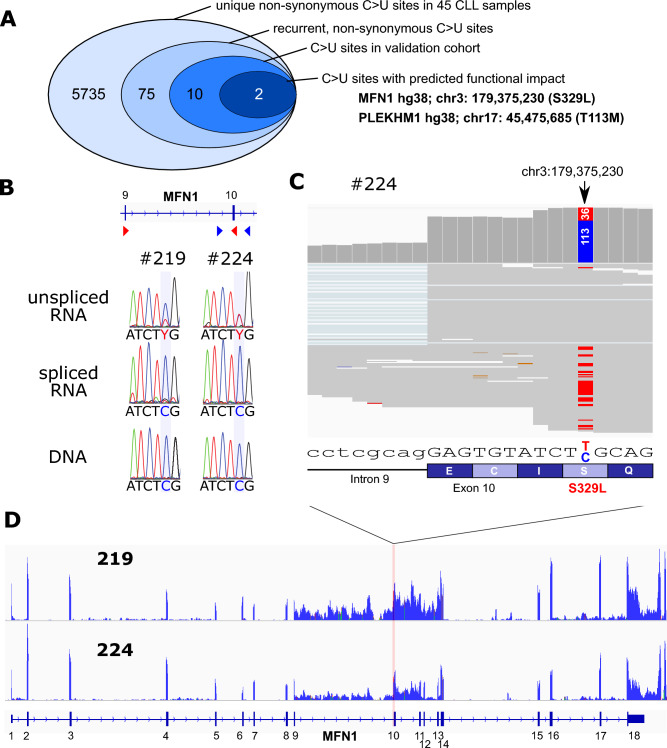
C > U editing in 2 CLL cohorts. (**A**) Extraction of recurrent C > U editing sites from 2 independent CLL cohorts. (**B**) Sanger sequencing confirms C > U editing in *MFN1* transcripts with retained intron 9 (Y denotes presence of a pyrimidine C or T). (**C**,** D**) Integrated genome viewer analysis of RNAseq reads mapping to *MFN1* at the C > U editing site (CLL patient #219 and #224).

We systematically analysed global C-to-U RNA editing in a previously documented cohort of 45 primary chronic lymphocytic leukemia patients (CLL cohort #1, NCBI-SRA accession-number PRJNA540189) by determining single base RNA/DNA differences from matched exome/transcriptome sequencing data^16,22^. From these 45 samples, we found a total of 5735 non-synonymous C > U differences, which comprised 75 recurrent (present in at least 2 samples), non-synonymous C > U editing events. We then validated these editing sites by screening RNA-seq data from a validation cohort of 98 samples (CLL cohort #2, EGA accession number EGAS00001000374)^23^, resulting in 10 remaining validated C > U editing sites, of which 2 showed predicted functional impact (Fig. 1A, Table S1). These 2 C > U sites comprised the genes *MFN1* (hg38; chr3:179,375,230; S329L) and *PLEKHM1* (hg38; chr17: 45,475,685; T113M). We next analysed C > U editing of these 2 sites by Sanger sequencing of genomic DNA and cDNA from CLL samples from our cohort (CLL cohort #1). While *PLEKHM1* editing could not be determined by Sanger sequencing (data not shown), we could clearly verify *MFN1* editing by Sanger sequencing in two samples with high *MFN1* editing from bioinformatic RNA/DNA sequencing data analysis (#219, #224, Fig. 1B). Strikingly, we found that C > U editing of *MFN1* was largely restricted to unspliced *MFN1* transcripts (Fig. 1B). This was consistent with our RNA-seq data from these two patients, in which we observed *MFN1* C > U editing in RNA-seq reads, mostly mapping to unspliced *MFN1* transcripts with retained intron 9 (Fig. 1C and D).

### MFN1 editing correlates with intron retention in different cell types

**Fig. 2:**
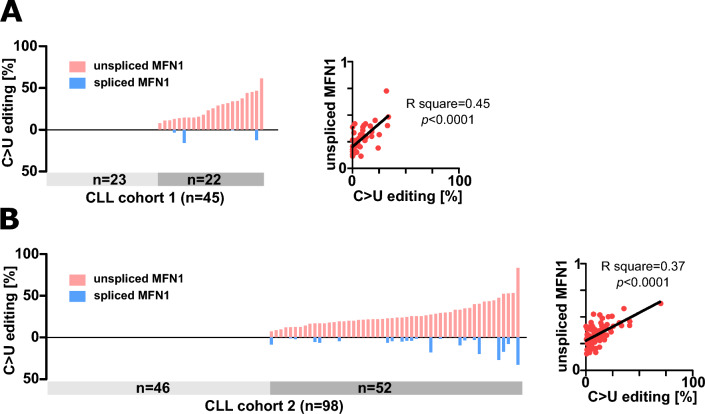
C > U editing is largely restricted to unspliced *MFN1*. Percentage of C > U editing in reads mapping to spliced or unspliced *MFN1* in CLL cohort #1 (**A**) and CLL cohort #2 (**B**). Correlation of C > U editing and *MFN1* splicing is shown on the right.

Next, we reanalyzed our RNA-seq data from both CLL cohorts for presence of C > U editing events in reads mapping to spliced or unspliced MFN1. Thereby, we detected a clear bias towards editing within the unspliced *MFN1* transcript although editing in spliced *MFN1*, which leads to S329L recoding, was observed in a few cases with IGVH mutated as well as unmutated IGHV (Fig. 2, Table S2). Moreover, we observed significant correlation of *MFN1* C > U editing depth (frequency of edited transcripts) and fraction of unspliced *MFN1* transcripts with intron 9 retention (Fig. 2, Table S2).

**Fig. 3:**
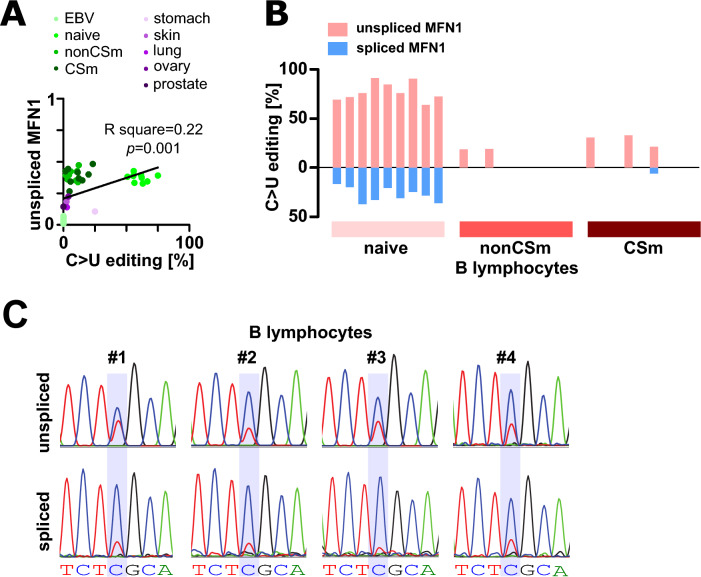
*MFN1* is robustly edited in B cells. (**A**) Correlation of C > U editing and *MFN1* splicing is shown for different cell types (EBV transformed B cells, naïve B cells, non class switched memory (nonCSm) B cells, class switched memory (CSm) B cells, stomach, skin, lung, ovary, prostate). (**B**) Percentage of C > U editing in reads mapping to spliced or unspliced *MFN1* in B cell subsets. (**C**) Sanger sequencing confirms C > U editing in *MFN1* transcripts in B cells from four different healthy donors.

We then aimed to analyse *MFN1* editing in healthy tissue. Therefore, we accessed publicly available RNA-seq datasets from stomach, skin, lung, ovary and prostate as well as from B cell subsets (EBV-transformed B cells, naïve, memory and class switched memory B cells) and extracted C > U editing depth at *MFN1* transcripts. Again, we found editing at variable depth in different tissues, with editing frequency correlating with retention of intron 9 (Fig. 3A, Table S3).

Surprisingly, substantial editing in mature spliced *MFN1* transcripts was observed in naïve B cells with S329L recoding frequencies between 17 and 38%, but not in class switched or non-class switched memory B cells (Fig. 3B). This was confirmed by Sanger sequencing of PCR products corresponding to spliced and unspliced *MFN1* amplified from cDNA of purified B cells from healthy donors (Fig. 3C).

### ADARB1 supports MFN1 editing

**Fig. 4:**
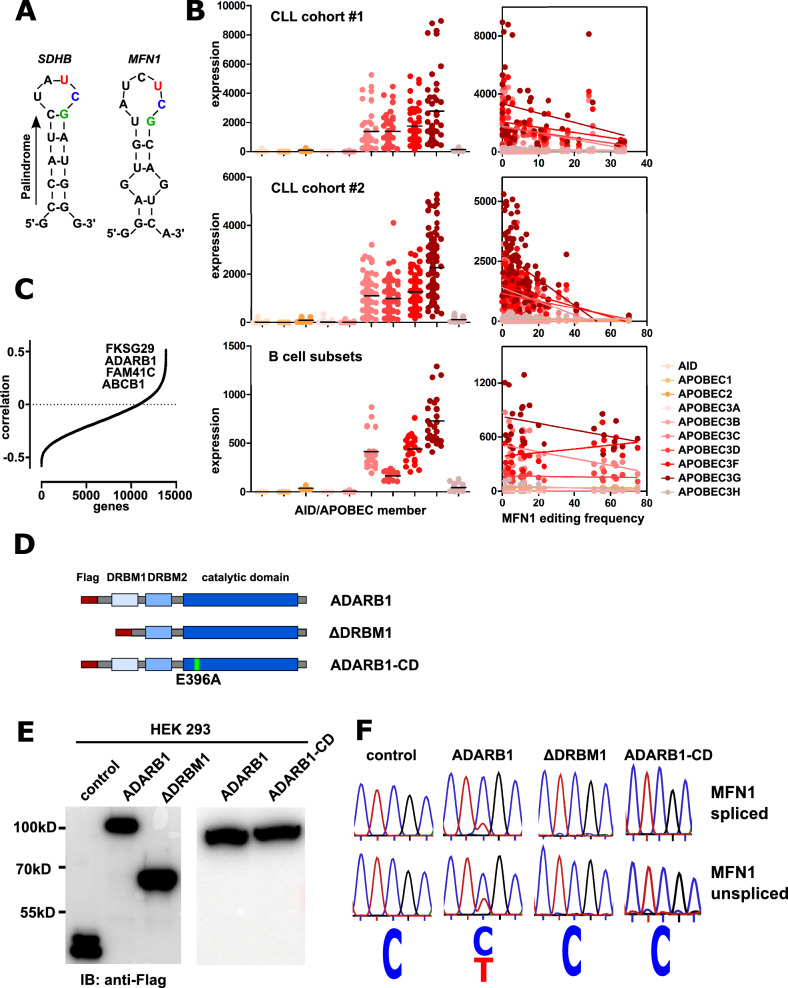
*MFN1* editing requires ADARB1. (**A**) *MFN1* editing site is located within a previously defined palindromic consensus motif present in *SDHB* transcripts. (**B**) Expression levels of AID/APOBEC members and correlation with C > U *MFN1* editing in two CLL cohorts and normal B cell subsets. (**C**) Mean values from a Pearson correlation from gene expression and *MFN1* editing from two CLL cohorts and B cell samples. The four top candidates positively correlating with editing are indicated in the graph. (**D**) Schematic representation of Flag-tagged ADARB1, ΔDRMB1 and ADARB1-CD constructs. (**E**) Western blot analysis of HEK293 cells transfected with the respective Flag-tagged constructs. (**F**) Representative Sanger sequencing of *MFN1* C-to-U editing sites from spliced and unspliced *MFN1* transcripts in HEK293 cells transfected with the respective Flag-tagged constructs.

Based on previous reports, we expected expression of the AID/APOBEC member responsible for *MFN1* editing to be associated with editing frequencies^10^. Although the *MFN1* editing site is located within a previously defined APOBEC3A consensus motif (5’-UCG-3’) flanked by palindromic sequences^10^ (Fig. 4A), *MFN1* editing frequencies in the CLL cohorts and normal B cells did not positively correlate with any *APOBEC* expression levels (Fig. 4B). To find candidates for putative positive editing regulators, we performed Pearson correlation analysis using editing frequency and RNA expression from these three cohorts. We screened the top candidates particularly for proteins with known RNA binding activity and found a positive correlation of *MFN1* editing frequency and expression levels of *ADARB1*, an A-to-I RNA editing enzyme (Fig. 4C). To test an influence of ADARB1 on *MFN1* editing, we cloned Flag-tagged *ADARB1* and a mutant lacking the dsRNA binding motif 1 (ΔDRBM1) together with a catalytically dead mutant ADARB1-CD, which has an E396A mutation^24^ (Fig. 4D). We transfected HEK293 cell lines with the different ADARB1 constructs and determined editing frequencies of endogenous *MFN1* transcripts. We found that transient transfection of *ADARB1* was sufficient to induce robust *MFN1* editing in HEK293 cell lines (Fig. 4E, F), showing that ADARB1 is indeed a factor supporting *MFN1* C-to-U editing. As expected, ΔDRBM1 did not support editing, as this mutant fails to bind RNA. However, surprisingly, also cells transfected with ADARB1-CD did not show editing of *MFN1*, indicating that the catalytic domain of ADARB1 is necessary for *MFN1* C-to-U editing (Fig. 4E, F). To fully clarify, whether also other ADARs can mediate this C-to-U editing event, we also cloned Flag-tagged versions of ADAR1 isoforms p110 and p150 and determined editing frequencies of endogenous *MFN1* transcripts in transfected HEK293 cells. Thereby we could confirm that only ADARB1 but not other ADAR1 isoforms is competent to induce C-to-U editing in *MFN1* transcripts (Fig. S1).

### MFN1 editing is mediated by APOBEC3 and is coupled to MFN1 intron retention

**Fig. 5:**
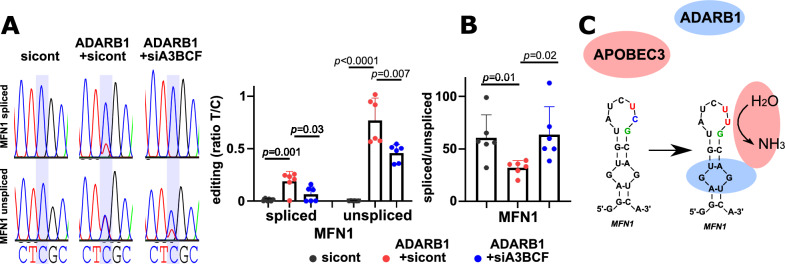
APOBEC3 proteins and ADARB1 cooperatively induce *MFN1* editing. (**A**) Representative Sanger sequencing of *MFN1* C-to-U editing sites from spliced and unspliced *MFN1* transcripts in HEK293 cells transfected with control siRNAs (sicont), siRNAs specific for APOBEC3B,C,F (siA3BCF) or Flag-tagged ADARB1 (ADARB1). Graph shows the statistics of editing frequencies from Sanger sequences from 6 independent experiments. (**B**) Ratio of spliced to unspliced *MFN1* transcripts determined by qPCR from experiments shown in (A). (**C**) A model illustrating ADARB1 dependent *MFN1* C-to-U editing by APOBEC3 deaminases. In this model, ADARB1 recruits (directly or indirectly) APOBEC3 proteins to *MFN1* pre-mRNAs, thereby enabling C-to-U deamination by APOBEC3 and interfering with splicing. In A and B, mean + SD is shown. Significances calculated using unpaired t-test from 6 independent experiments.

To detect potential transcriptome-wide ADARB1 dependent C-to-U editing events, we performed RNA-seq from ADARB1 and control-transfected HEK293 cells and called specific editing events. In line to previous reports, we detected many ADARB1 induced A-to-I editing events^1^ in two independent experiments, whereas C-to-U editing was exclusively restricted to *MFN1*, showing that this is the sole ADARB1 dependent C-to-U editing site present in RNA from 293HEK cells (RNA editing data from RNAseq and small RNA seq depicted in Table S4, S5). However, RNA-seq analysis revealed many different A-to-I editing sites clustering around the C-to-U site within *MFN1*. Each of the A-to-I editing sites were low frequent and most of them were non-recoding events (Fig S2). Interestingly, four of these A-to-I editing sites, which are closest to the C-to-U editing site were also present in CLL and B cell samples (A-to-I editing sites at position −4, + 3, + 7 and + 10 relative to the C-to-U site) and correlated with C-to-U editing (Fig S2B). Hence, these A-to-I events support the concept of direct binding of ADARB1 near to the *MFN1* C-to-U editing site. Next, to determine, which cytidine deaminase executes *MFN1* C-to-U editing, we extracted expression levels of AID/APOBEC cytidine/cytosine deaminase family members from the RNA-seq data. From the catalytically active AID/APOEBC members, we did not find transcripts for *AID, A1, A2 and A3A* and only very low expression levels for *A3D* and *H*, pointing to *A3B, C or F* as the likely candidates for catalyzing *MFN1* C-to-U editing (Fig S3, Table S6). To assess whether *MFN1* editing is attributed to C-to-U deamination by *A3B, C* or* F*, we performed pooled knockdown experiments of *A3B, C* and *F* using small interfering RNAs (siRNAs) in HEK293 cells together with *ADARB1* transfection. Indeed, we observed a decrease in *MFN1* editing frequencies in ADARB1 transfected cells upon *A3BCF* knockdown compared to control siRNAs, both in spliced as well as unspliced *MFN1* transcripts (Fig. 5A and Fig S4). Notably, also individual knockdowns of A3B, C or F led to decreased C-to-U editing while combined A3BCF knockdown had the biggest impact on reduction of editing frequencies (Fig S5). To examine the interrelation of *MFN1* editing and *MFN1* intron retention, we established a taqman based qPCR assay to specifically determine transcript levels of *MFN1* with or without retained intron 9. Although most transcripts remained fully spliced, we found that *ADARB1* expression slightly increased *MFN1* intron retention, which was not observed upon simultaneous knockdown of *A3BCF* (Fig. 5B). Hence, our data promote a model in which ADARB1 and APOBEC3 cooperate to highly specifically induce C-to-U editing and intron retention of *MFN1* transcripts to regulate *MFN1* abundance and S329L recoding (Fig. 5C).

### MFN1 editing and CLL disease course

**Fig. 6:**
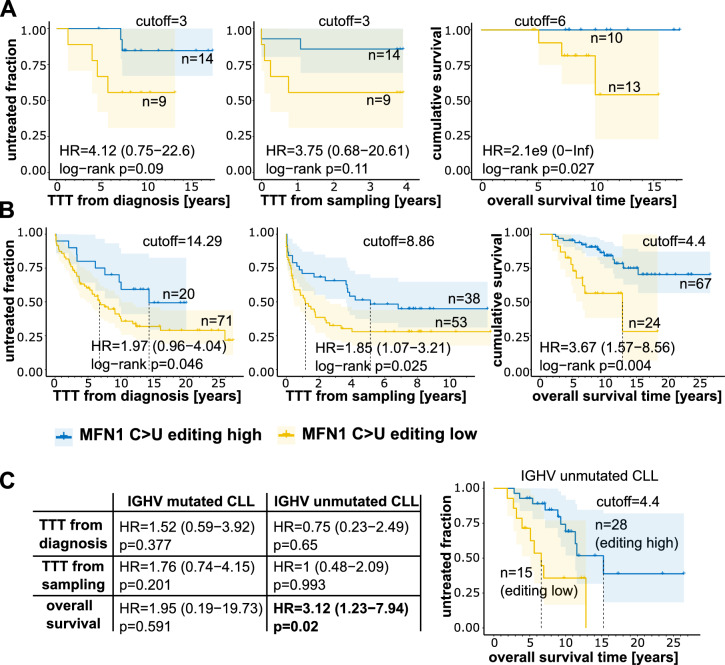
*MFN1* editing associates with prolonged survival. Kaplan–Meier survival plots show time to treatment (TTT) from diagnosis, from sampling and overall survival in the pilot cohort (**A**) and the validation cohort (**B**). Hazard ratios (HR) with confidence intervals (CI), log-rank p-values, cut-offs (%*MFN1* editing) and sample size (n) are indicated within the graphs. (**C**) TTT and OS in the CLL validation cohort according to IGHV mutation status (cutoffs used as in B).

MFN1 S329L recoding was previously shown to critically affect mitochondrial fusion and, thus, in turn impacts mitochondria-dependent cellular homeostasis and apoptosis^19^. This prompted us to evaluate an association of *MFN1* editing on the course of disease in CLL patients. Therefore, we analysed a pilot cohort of 23 CLL patients, where sampling was done during indolent disease stage (table S7). From this cohort, we performed RNA-seq from CLL samples and determined *MFN1* editing frequencies. Cox-regression analysis and log-rank testing revealed that high frequency of *MFN1* C-to-U editing associated with prolonged treatment-free survival (time to treatment, TTT) from time of diagnosis as well as sampling (Fig. 6A). In addition, overall survival (OS) was significantly prolonged in patients with high frequency *MFN1* editing (Fig. 6A). To corroborate these findings, we analysed *MFN1* editing and course of disease in the validation cohort (CLL cohort #2) of which RNA-seq and clinical data were available^23^. In line with findings from the pilot cohort, we observed significantly prolonged TTT as well as OS in patients with high *MFN1* editing frequencies (Fig. 6B). While TTT was not significantly different within the IGHV mutated or unmutated samples, OS remained significantly prolonged in IGHV-unmutated CLL patients with high *MFN1* editing (Fig. 6C). Gene expression analysis from RNAseq data revealed that ADARB1 was highly significantly upregulated in *MFN1*-editing-high samples from the CLL validation cohort. APOBEC3 gene members did not show significant upregulation in *MFN1*-editing-high samples (supplementary Fig S7). Of note, *MFN2* was slightly upregulated in the editing-high group, while *MFN1* levels were similar. However, transcript levels of C-to-U edited *MFN1* were significantly higher in the editing-high group (Fig S7). Finally, gene ontology (GO) term analysis of RNAseq data revealed that many biological pathways were significantly deregulated between editing high and low groups, including those mapping to RNA metabolisms (splicing, processing). Pathways assigned to cell death or mitochondrial functions were not found significantly deregulated (Table S8).

## Discussion

In this study, we show for the first time that the A-to-I editing enzyme ADARB1 plays a novel role in C-to-U RNA editing. Particularly, we found that A3BCF together with ADARB1 cooperate to induce C-to-U editing at *MFN1* transcripts, thereby interfering with *MFN1* splicing and mediating MFN1 S329L conversion, with robust S329L recoding primarily occurring in naïve B lymphocytes and a subset of primary CLL cells. Although ADARB1 has been previously reported to mediate many A-to-I editing events and to regulate RNA splicing, ADARB1 has so far not been implicated in C-to-U editing^25,26^. Interestingly, catalytically inactive ADARB1 E396A mutants were not competent for APOBEC3 mediated C-to-U editing of the *MFN1* transcripts. Mechanistically, several models for *MFN1* C-to-U editing can be brought forward: first, the catalytically dead ADARB1 mutant might be compromised in its ability to form a complex with *MFN1*-RNA substrate and the APOBEC3 enzyme. However, we consider this possibility rather unlikely, as the ADARB1 E396A mutant has been shown to retain full RNA binding capacities^27^. Moreover, a catalytically inactive variant would possibly bind with even higher affinity to RNA targets as A-to-I deamination likely promotes dissociation of ADARB1 from the RNA substrate. Second, ADARB1 might be able to catalyze the C-to-U deamination directly due to a rare and unique conformation of the flipped out cytidine. However, we also consider this possibility rather unlikely, because the C-to-U deamination can be reduced by knockdown of APOBEC3, pointing to APOBEC3 members to catalyze the C-to-U deamination. Third, inactive ADARB1 might lose processivity on RNA targets, which could be necessary for bound APOBEC3 to come in proximity to the C-to-U editing site.

Alternatively, ADARB1 binding to and editing of *MFN1* transcripts could alter the secondary RNA structure, allowing recruitment of A3 members. These possibilities are not mutually exclusive and more research will be necessary to fully delineate the mechanism behind *MFN1*-editing. However, the scattered low-level A-to-I editing events nearby the *MFN1* C-to-U editing site correlate with C-to-U editing, which we interpret as footprint of ADARB1 activity and as evidence that ADARB1 is physically interacting with *MFN1* transcripts in proximity to the C-to-U editing site. Furthermore, our data show that *MFN1* editing is associated with prolonged treatment-free and overall survival in CLL patients. During our study on CLL, another team reported S329L MFN1 editing in brain samples and hypoediting of this site in samples from schizophrenia patients^19^. There, S329L recoding was shown to affect the function of the MFN1 encoding protein mitofusin-1. Mitofusin-1 is an important protein mediating mitochondrial fusion and network formation, which alters a diverse set of physiological processes such as mitochondria-dependent cell death, calcium homoeostasis, energy metabolism and proliferation^28–30^. MFN1 protein, as well as the homolog MFN2 consists of a GTPase domain, two coiled-coil heptad repeat domains (HR1 and HR2) and a transmembrane domain with the amino and carboxy termini both facing into the cytosol. GTP hydrolysis enables mitochondrial fusion upon interaction of two opposing HR2 domains from homo- or hetero-oligomeric complexes (MFN1-MFN1, MFN2-MFN2 or MFN1-MFN2 complexes)^31^. Although S329 is not directly located within one of the known MFN1 domains but between the GTPase and the HR1 domain, S329L conversion was shown to perturb MFN1 function in mitochondrial fusion and to increase cellular apoptosis^19^. Decreased cell fitness upon S329L recoding in leukemic cells would in turn alleviate the disease, which fits to our observed prolonged survival in CLL patients with high *MFN1*-editing frequencies. Nonetheless, a possible benefit of S329L recoding for the respective CLL cells and for naïve B cells remains to be investigated, which likely reflects the complex relationship between mitochondrial morphology and cellular homeostasis in different cell types. Of note, pathway analysis revealed a couple of differentially regulated biological pathways in CLL groups with high versus low *MFN1*-editing, however, we could not find deregulated pathways related to mitochondria dependent functions or cell death regulation.

While it was originally reported that APOBEC2 mediates S329L editing of *MFN1* in brain samples^19^, our data suggest that in B cells and CLL cells APOBEC3 family members seem to be responsible for this editing event, as APOBEC2 was not expressed in the cells analysed in our study. This could mean that several APOBEC enzymes can redundantly perform this specific editing, depending on which of them are predominantly present.

Our data further show that S329L recoding is associated with intron retention, which has not been reported so far. Hence, *MFN1* editing and intron retention regulates availability of mature *MFN1* transcripts and in turn MFN1 protein levels. This is particularly interesting in light of the fact that sole MFN1 levels critically affect mitochondrial fusion and network formation^28,32^.

Notably, the extent of intron retention observed in ADARB1 transfected HEK293 cell lines was much less than in ADARB1 positive CLL cells, despite pronounced *MFN1* editing, indicating that additional factors may contribute to intron retention. Our data are especially interesting considering the fact that resistance to current therapies with BCL2 inhibitors, which promote mitochondria dependent cell death in CLL is also associated with perturbed mitochondria morphologies^33^. Hence, a possible role of *MFN1* editing might also be attributed in context of cancer drug resistance, which will be interesting to elucidate in future studies. Since fusion of mitochondria is critical for a diverse set of pathways, *MFN1* editing may be a novel and thus far unexplored mechanism regulating or fine-tuning a diverse set of mitochondria-dependent cellular processes in cancer^29,31^. In light of the data from our study and the fact that perturbed mitochondrial network dynamics is frequently associated with several disease states such as cancer, diabetes or neurodegeneration^29,30,34^, aberrant *MFN1* editing might contribute to the pathophysiology of these diseases, warranting a more thorough investigation of *MFN1* editing in future studies.

## Methods

### Patients

RNA/DNA sequencing data from CLL samples and B cell subsets were downloaded from accession-number SUB5459211 (https://www.ncbi.nlm.nih.gov/bioproject/PRJNA540189)^22^ and (EGAS00001000374)^23^. In addition, RNA sequencing data of normal tissue were downloaded from the European Genome-phenome Archive and the ENCODE database https://www.encodeproject.org/ (ENCSR000AFG, ENCSR000AFC, ENCSR042GYH, ENCSR495HDM, ENCSR853WOM). For Kaplan–Meier analyses of a pilot CLL cohort, peripheral blood from 23 chemo naïve CLL patients was collected upon informed consent and ethical approval by the Ethics Committee of the Province of Salzburg (415-E/1287/4–2011, 415-E/1287/8–2011, 415-E/1287/20–2018) and was conducted in accordance with the ethical principles outlined in the Declaration of Helsinki. CLL cells were obtained by density gradient centrifugation and the B-CLL Cell Isolation kit (Miltenyi Biotec). Cell purity was > 90% in all samples. The assessment of prognostic markers was performed routinely at our department as described previously^35^. B cells from healthy donors were isolated from peripheral blood using magnetic beads (B cell isolation kit, Miltenyi Biotec) (ethics number EP/73/791). RNA was purified using RNeasy Mini kit (Qiagen).

### Calling RNA editing events

Matched Exome and Transcriptome bam-files were sorted after alignment to hg38 with bowtie2 and tophat2, respectively, and duplicates were marked by picard-tools (v2.2.2, https://broadinstitute.github.io/picard/). The processed bam files were processed using samtools-0.1.19^36^ mpileup and loci with a mean base quality phred score below 20 or with a coverage of less than 6 reads in either DNA or RNA were omitted. We then used a custom perl script to remove variants that occurred in the first or last base of a read or were strand-biased (present only in either forward or reverse reads). We furthermore omitted variants in deleted or inserted regions and selected only variants uniquely found in RNA by using the somatic variant caller VarScan2 (–min-coverage 8 –min-var-freq 0.05 –strand-filter 1), filtered for high confidence variants (processSomatic, see Koboldt et al^37^, “Basic Protocol 2”) and subsequently annotated them using ANNOVAR^38^. A custom perl script and information from hg19.refseq.bed file retrieved from the UCSC database were used to translate variants in genes with reverse strand orientation. Variants in HLA genes were excluded. Functional impact of editing sites was predicted using mutationassessor (mutationassessor.org, accessed February 2019) (Reva et al. 2011).

### Calculating MFN1 splicing and editing levels

Downloaded fastq files were aligned to the human hg19 reference genome using the STAR aligner^39^ (using default options and –outFilterMultimapScoreRange 1). MFN1 intron/exon intervals (chr3:179,085,824–179,085,891|Exon_9;chr3:179,085,892–179,093,007|Intron_9;chr3:179,093,008–179,093,129|Exon_10;chr3:179,093,130–179,094,829|Intron_10;chr3:179,094,830–179,094,956|Exon_11) were saved in bed format (MFN1_exons.bed). GATK DepthOfCoverage (v3.7.0^40^) was used to assess coverage at the indicated introns/exons (-L MFN1_exons.bed, -U ALLOW_N_CIGAR_READS).

Samtools (v1.9) view was used to filter reads aligned to the 5’ intron/exon border at exon 10 (chr3:179,093,008–179,093,009) and spliced and unspliced reads were separated using “awk ‘($6 ~/N/)’” or “awk ‘($6! ~/N/)’”, respectively. The resulting files were reconverted to the bam format and variants between spliced and unspliced reads were assessed using samtools mpileup (-r"chr3:179,093,018–179,093,018"and varscan mpileup2snp (–min-reads2 1 –min-var-freq 0.001).

### RNA sequencing of transfected HEK293 cells

For RNA sequencing, DNA-free total RNA (300 ng as input) quantified by Qubit 2.0 (Life technologies) and quality checked on TapeStation 4200 (Agilent) was used. The libraries were prepared following NEBNext Poly(A) mRNA Magnetic Isolation Module of Ultra II Directional RNA Library Prep Kit for Illumina (NEB). Libraries were pooled according to the ratio desired read number/number of samples. Denaturation and dilution was conducted following the standard normalization method for NextSeq 500 and NextSeq 550 Sequencing Systems (Illumina), with 1% PhiX library as a sequencing control. The libraries were then sequenced from both ends (paired-end) on Illumina NextSeq500/550 platform with 2 × 150 cycles. Analysis of editing events was performed as described above.

For small RNA sequencing, total RNA was isolated from transfected samples, using Monarch Total RNA Miniprep Kit (NEB) according to the protocol for cultured mammalian cells. The NEBNext Multiplex Small RNA Library Prep Set for Illumina (NEB) was adapted to convert small RNAs into indexed libraries. The final libraries were diluted to 10 pM according to the recommended standard normalization method for MiSeq System (Illumina). Libraries were sequenced using MiSeq Reagent kit V2 with 50 cycles and 1% PhiX as a sequencing control. Fastq files were trimmed using cutadapt (^41^ GATCGGAAGAGCACACGTCTGAACTCCAGTCAC, min = 18, max = 300, min_quality = 25) and aligned to hg38 using bowtie1^42^. Samtools^36^ mpileup (-B -q 1 -d 100,000) files were generated using the following sample-pairs: siControl-ADA_siControl, siControl-ADA_siBCF2, ADA_siControl-ADA_siBCF2. Edited variants were identified using varscan v2.4.4^43,44^, somatic –min-var-freq 0.05; processSomatic –min-tumor-freq 0.01 –max-normal-freq 0.05 –p-value 0.05; somaticFilter –min-var-freq 0.01). bam-readcount (https://github.com/genome/bam-readcount) was used to calculate read depth in variant sites and varscan fpfilter (–dream3-settings 1, –min-var-freq 0.01, –min-var-avgrl 12 min-ref-avgrl 15) was applied to identify high quality variants. Variants with somatic score (SSC) of 30 or higher were considered high quality edited sites. All sequencing data were uploaded to the Sequence Read Archive (SRA; https://www.ncbi.nlm.nih.gov/sra) SubmissionID: SUB11167983; BioProject ID: PRJNA814016.

### Plasmids and cloning

ADARB1 (encoding 701 amino acids, transcript variant 1, mRNA from RefSeq NM_001112.3) and ΔDRBM1 (lacking amino acid residues 5–196) expressing vectors were generated by PCR amplification of the respective ADARB1 variants from an ADARB1 cDNA-ORF clone (purchased from Sinobiologicals cat# HG18501-UT-1) using primers RG1800/RG1801 or RG1802/RG1801. The backbone of a Flag-tag expression plasmid was PCR-amplified using primers RG1798/RG1799 and fused with inserts using In-Fusion HD cloning (Takara Clontech). ADARB1-CD (catalytically dead variant by mutating E396A) was generated by in vitro mutagenesis using primers RG1841/RG1842. ADAR1 p110 and p150 isoforms were PCR amplified from human cDNA using primers RG2267/RG2268 and RG2269/RG2270 and cloned into the Flag-tag expression plasmid. For control, empty Flag-plasmids (generated by PCR-amplification of Flag-ADARB1 constructs using primers RG1820/RG1821 followed by In-Fusion HD-cloning) or constructs expressing an irrelevant protein (FLAG-RNF126 vector, generated by BamHI/HindIII cloning of PCR-amplified RNF126 from human cDNA using primers RG622/RG623 into a Flag-expression plasmid) were used. The Flag-expression plasmid was a kind gift from Silvestro Conticello^45^. Primers are listed in Table S9.

### Cell culture and treatments

HEK293 cells were cultured in RPMI medium (Gibco, ThermoFisher Scientific) supplemented with Fetal Bovine Serum (FBS; 10%), L-glutamine (1%), and Penicillin/Streptomycin (1%). Transfection was carried out using GeneJuice (Merck KGaA, Darmstadt, Germany) according to manufactures instructions. After 48 h, cells were harvested for several experiments.

To knockdown APOBEC family members, siRNAs were purchased from Dharmacon Company using On-TARGETplus technology (individual siRNAs or SMARTpool); a non-targeting siRNA (Table S10) was used as control (siCont). 300,000 cells at 95% confluence were treated with 20 pmol siRNAs using 6ul or 9ul Lipofectamine RNAimax (ThermoFisher Scientific). After 24 h, further transfections with expression plasmids were performed, and 72 h later total RNA was extracted for downstream experiments.

### Assessing editing frequency by Sanger sequencing

RNA was extracted from treated cells using High Pure RNA isolation kit (Roche). First strand complementary DNA (cDNA; 500 ng input RNA) was prepared using thermostable Luna Reverse Transcriptase (LunaScript RT SuperMix Kit, NEB) following manufacturer´s instructions. Editing sites were PCR-amplified from cDNA using Phusion High–Fidelity DNA Polymerase (ThermoFisher Scientific) using primers RG1464/RG1465 for unspliced (Un), and primers RG1462/RG1475 for spliced (Sp) MFN1. PCR products were gel purified (Macherey–Nagel) and Sanger sequenced (Eurofins, Germany). The Poly Peak Parser Sangerseq R package^46^ was applied to determine the peak amplitude for each base within a basecall window (peakAmpMatrix) from Sanger sequences. T to C ratio was calculated from the ratio of peak amplitude numbers. Primer sequences are listed in Table S8.

### Quantitative PCR

Transcript levels of spliced/unspliced MFN1 and APOBECs upon knockdown of APOBECs or overexpression of ADARB1 were assessed by Quantitative PCR (qPCR) using Luna Universal Probe qPCR Master Mix (NEB) on cDNA. Probes for APOBECs and GAPDH were purchased from ThermoFisherScientific. Customized probe (RG1810) and primers for unspliced (RG1464/1465) or spliced (RG1462/1475) MFN1 transcripts were purchased from Eurofins (Ebersberg, Germany) and listed in Table S8. Samples were processed on a ViiA 7 Real-Time PCR system (Applied Biosystems) with GAPDH as internal control, and the comparative C_T_ method was used for the final evaluation.

### Western blot analysis

Cells were harvested in PBS and lysed with RIPA buffer including Protease inhibitor cocktail (Roche) and PMSF for 30 min on ice and vortexing. After centrifugation (10 min, 14000 rpm, at 4 °C), protein concentration was determined applying Pierce BCA Protein assay kit (ThermoFisher Scientific) on Spark multimode reader (Tecan, Trading AG, Switzerland). Protein samples were prepared with 3 × Laemmli and RIPA buffer. Total protein (15 μg) of whole-cell extracts were loaded on 10% bis-acrylamide gel by Sodium Sodecyl Sulfate–polyacrylamide gel electrophoresis (SDS-PAGE). Expression of FLAG-tagged proteins in HEK293 transfectants was detected by Western blotting using HRP-conjugated anti-FLAG antibody M2 (Sigma-Aldrich Co. LLC), and anti-alpha tubulin antibody (GeneTex) as an internal control. Visualization of protein bands was done on Fusion FX (Vilber) using Amersham™ ECL Prime kit (GE Healthcare). Uncropped full length blots are provided as Fig S4.

### Statistical analysis

Statistical analyses were performed using Graph Pad Prism Version 10 (GraphPad Software, Inc.) or R (survminer and survival R packages) using statistical tests indicated in the respective figure legends. Cutt-offs for Kaplan–Meier graphs were calculated using the maximally selected rank statistics from the ‘maxstat’ R package, providing a value of a cutpoint that corresponds to the most significant relation with outcome. Differential gene expression analysis was performed using the „edgeR “ R package^47^. Editing high and low was defined by a cutoff of 14.29% editing frequency. DE genes were ranked by logFC * -log10(p-value) and subjected to gene ontology pathway analysis using the R package clusterProfiler^48^. No statistical analyses for sample size estimates were used. No blinding or randomization was used. All available samples from the CLL cohorts were included in the study. Sample sizes, statistical testing, are indicated in each figure legend. All figures were finalized using Inkscape 1.3.2.

## Supplementary Information

Supplementary Information 1.

Supplementary Information 2.

Supplementary Information 3.

Supplementary Information 4.

Supplementary Information 5.

Supplementary Information 6.

Supplementary Information 7.

Supplementary Information 8.

Supplementary Information 9.

Supplementary Information 10.

Supplementary Information 11.

## Data Availability

All sequencing data were uploaded to the Sequence Read Archive (SRA; https://www.ncbi.nlm.nih.gov/sra) accession PRJNA814016 and PRJNA1016100.

## References

[1] Eisenberg, E. & Levanon, E. Y. A-to-I RNA editing - immune protector and transcriptome diversifier. *Nat. Rev. Genet* **19**, 473–490. 10.1038/s41576-018-0006-1 (2018). 29692414 10.1038/s41576-018-0006-1

[2] Bazak, L. et al. A-to-I RNA editing occurs at over a hundred million genomic sites, located in a majority of human genes. *Genome Res.* **24**, 365–376. 10.1101/gr.164749.113 (2014). 24347612 10.1101/gr.164749.113 PMC3941102

[3] Levanon, E. Y., Cohen-Fultheim, R. & Eisenberg, E. In search of critical dsRNA targets of ADAR1. *Trend. Genet* **40**, 250–259. 10.1016/j.tig.2023.12.002 (2024). 10.1016/j.tig.2023.12.002 38160061

[4] Rebhandl, S., Huemer, M., Greil, R. & Geisberger, R. AID/APOBEC deaminases and cancer. *Oncoscience*10.18632/oncoscience.155 (2015). 26097867 10.18632/oncoscience.155 PMC4468319

[5] Okuyama, S. et al. Excessive activity of apolipoprotein B mRNA editing enzyme catalytic polypeptide 2 (APOBEC2) contributes to liver and lung tumorigenesis. *Int. J. Cancer* **130**, 1294–1301. 10.1002/ijc.26114 (2012). 21469143 10.1002/ijc.26114

[6] Teng, B., Burant, C. F. & Davidson, N. O. Molecular cloning of an apolipoprotein B messenger RNA editing protein. *Science* **260**, 1816–1819. 10.1126/science.8511591 (1993). 8511591 10.1126/science.8511591

[7] Rosenberg, B. R., Hamilton, C. E., Mwangi, M. M., Dewell, S. & Papavasiliou, F. N. Transcriptome-wide sequencing reveals numerous APOBEC1 mRNA-editing targets in transcript 3’ UTRs. *Nat. Struct. Mol. Biol.* **18**, 230–236. 10.1038/nsmb.1975 (2011). 21258325 10.1038/nsmb.1975 PMC3075553

[8] Blanc, V. et al. Genome-wide identification and functional analysis of Apobec-1-mediated C-to-U RNA editing in mouse small intestine and liver. *Genome Biol.* **15**, R79. 10.1186/gb-2014-15-6-r79 (2014). 24946870 10.1186/gb-2014-15-6-r79 PMC4197816

[9] Sato, Y. et al. Deficiency in APOBEC2 leads to a shift in muscle fiber type, diminished body mass, and myopathy. *J. Biol. Chem.* **285**, 7111–7118. 10.1074/jbc.M109.052977 (2010). 20022958 10.1074/jbc.M109.052977 PMC2844160

[10] Sharma, S. et al. APOBEC3A cytidine deaminase induces RNA editing in monocytes and macrophages. *Nat. Commun.* **6**, 6881. 10.1038/ncomms7881 (2015). 25898173 10.1038/ncomms7881 PMC4411297

[11] Sharma, S., Patnaik, S. K., Taggart, R. T. & Baysal, B. E. The double-domain cytidine deaminase APOBEC3G is a cellular site-specific RNA editing enzyme. *Sci. Rep.* **6**, 39100. 10.1038/srep39100 (2016). 27974822 10.1038/srep39100 PMC5156925

[12] Sharma, S. & Baysal, B. E. Stem-loop structure preference for site-specific RNA editing by APOBEC3A and APOBEC3G. *PeerJ* **5**, e4136. 10.7717/peerj.4136 (2017). 29230368 10.7717/peerj.4136 PMC5723131

[13] Sharma, S., Patnaik, S. K., Kemer, Z. & Baysal, B. E. Transient overexpression of exogenous APOBEC3A causes C-to-U RNA editing of thousands of genes. *RNA Biol.* **14**, 603–610. 10.1080/15476286.2016.1184387 (2017). 27149507 10.1080/15476286.2016.1184387 PMC5449087

[14] Romano, G. et al. Non-coding RNA editing in cancer pathogenesis. *Cancers (Basel)*10.3390/cancers12071845 (2020). 32650588 10.3390/cancers12071845 PMC7408896

[15] Pu, S., Cheng, T. & Cheng, H. Advances in RNA editing in hematopoiesis and associated malignancies. *Blood*10.1182/blood.2024027379 (2025). 39869834 10.1182/blood.2024027379

[16] Gassner, F. J. et al. RNA editing contributes to epitranscriptome diversity in chronic lymphocytic leukemia. *Leukemia* **35**, 1053–1063. 10.1038/s41375-020-0995-6 (2021). 32728184 10.1038/s41375-020-0995-6 PMC8024191

[17] Gassner, F. J., Zaborsky, N., Feldbacher, D., Greil, R. & Geisberger, R. RNA editing alters miRNA Function in chronic lymphocytic leukemia. *Cancers (Basel)*10.3390/cancers12051159 (2020). 32380696 10.3390/cancers12051159 PMC7280959

[18] Santel, A. & Fuller, M. T. Control of mitochondrial morphology by a human mitofusin. *J. Cell Sci.* **114**, 867–874. 10.1242/jcs.114.5.867 (2001). 11181170 10.1242/jcs.114.5.867

[19] Choudhury, M. et al. Widespread RNA hypoediting in schizophrenia and its relevance to mitochondrial function. *Sci. Adv.* **9**, eade9997. 10.1126/sciadv.ade9997 (2023). 37027465 10.1126/sciadv.ade9997 PMC10081846

[20] Simula, L., Nazio, F. & Campello, S. The mitochondrial dynamics in cancer and immune-surveillance. *Semin. Cancer Biol.* **47**, 29–42. 10.1016/j.semcancer.2017.06.007 (2017). 28655520 10.1016/j.semcancer.2017.06.007

[21] Tabara, L. C., Segawa, M. & Prudent, J. Molecular mechanisms of mitochondrial dynamics. *Nat. Rev. Mol. Cell Biol.* **26**, 123–146. 10.1038/s41580-024-00785-1 (2025). 39420231 10.1038/s41580-024-00785-1

[22] Egle, A. et al. Fludarabine and rituximab with escalating doses of lenalidomide followed by lenalidomide/rituximab maintenance in previously untreated chronic lymphocytic leukaemia (CLL): the REVLIRIT CLL-5 AGMT phase I/II study. *Ann. Hematol.*10.1007/s00277-018-3380-z (2018). 29862437 10.1007/s00277-018-3380-z PMC6097797

[23] Ferreira, P. G. et al. Transcriptome characterization by RNA sequencing identifies a major molecular and clinical subdivision in chronic lymphocytic leukemia. *Genome Res.* **24**, 212–226. 10.1101/gr.152132.112 (2014). 24265505 10.1101/gr.152132.112 PMC3912412

[24] Singh, M. et al. Hyperphagia-mediated obesity in transgenic mice misexpressing the RNA-editing enzyme ADAR2. *J. Biol. Chem.* **282**, 22448–22459. 10.1074/jbc.M700265200 (2007). 17567573 10.1074/jbc.M700265200

[25] Sommer, B., Kohler, M., Sprengel, R. & Seeburg, P. H. RNA editing in brain controls a determinant of ion flow in glutamate-gated channels. *Cell* **67**, 11–19. 10.1016/0092-8674(91)90568-j (1991). 1717158 10.1016/0092-8674(91)90568-j

[26] Tang, S. J. et al. Cis- and trans-regulations of pre-mRNA splicing by RNA editing enzymes influence cancer development. *Nat. Commun.* **11**, 799. 10.1038/s41467-020-14621-5 (2020). 32034135 10.1038/s41467-020-14621-5 PMC7005744

[27] Valente, L. & Nishikura, K. RNA binding-independent dimerization of adenosine deaminases acting on RNA and dominant negative effects of nonfunctional subunits on dimer functions. *J. Biol. Chem.* **282**, 16054–16061. 10.1074/jbc.M611392200 (2007). 17428802 10.1074/jbc.M611392200 PMC2954279

[28] Park, Y. Y., Nguyen, O. T., Kang, H. & Cho, H. MARCH5-mediated quality control on acetylated Mfn1 facilitates mitochondrial homeostasis and cell survival. *Cell Death Dis.* **5**, e1172. 10.1038/cddis.2014.142 (2014). 24722297 10.1038/cddis.2014.142 PMC5424118

[29] Xie, L. L. et al. Mitochondrial network structure homeostasis and cell death. *Cancer Sci.* **109**, 3686–3694. 10.1111/cas.13830 (2018). 30312515 10.1111/cas.13830 PMC6272111

[30] Dai, W. & Jiang, L. Dysregulated mitochondrial dynamics and metabolism in Obesity, Diabetes, and Cancer. *Front. Endocrinol. (Lausanne)* **10**, 570. 10.3389/fendo.2019.00570 (2019). 31551926 10.3389/fendo.2019.00570 PMC6734166

[31] Chandhok, G., Lazarou, M. & Neumann, B. Structure, function, and regulation of mitofusin-2 in health and disease. *Biol. Rev. Camb. Philos. Soc.* **93**, 933–949. 10.1111/brv.12378 (2018). 29068134 10.1111/brv.12378 PMC6446723

[32] Suen, D. F., Norris, K. L. & Youle, R. J. Mitochondrial dynamics and apoptosis. *Genes. Dev.* **22**, 1577–1590. 10.1101/gad.1658508 (2008). 18559474 10.1101/gad.1658508 PMC2732420

[33] Thomalla, D. et al. Deregulation and epigenetic modification of BCL2-family genes cause resistance to venetoclax in hematologic malignancies. *Blood* **140**, 2113–2126. 10.1182/blood.2021014304 (2022). 35704690 10.1182/blood.2021014304 PMC10653032

[34] Lou, G. et al. Mitophagy and neuroprotection. *Trend. Mol. Med.*10.1016/j.molmed.2019.07.002 (2019). 10.1016/j.molmed.2019.07.002 31375365

[35] Gassner, F. J. et al. Chemotherapy-induced augmentation of T cells expressing inhibitory receptors is reversed by treatment with lenalidomide in chronic lymphocytic leukemia. *Haematologica* **99**, 67–69. 10.3324/haematol.2013.098459 (2014). 24561794 10.3324/haematol.2013.098459 PMC4008106

[36] Li, H. et al. The sequence alignment/map format and SAMtools. *Bioinformatics* **25**, 2078–2079 (2009). 19505943 10.1093/bioinformatics/btp352 PMC2723002

[37] Koboldt, D. C., Larson, D. E. & Wilson, R. K. Using VarScan 2 for germline variant calling and somatic mutation detection. *Curr. Protoc. Bioinform.* **44**, 15–17 (2013). 10.1002/0471250953.bi1504s44 PMC4278659 25553206

[38] Wang, K., Li, M. & Hakonarson, H. ANNOVAR: Functional annotation of genetic variants from high-throughput sequencing data. *Nucl. Acid. Res.* **38**, e164. 10.1093/nar/gkq603 (2010). 10.1093/nar/gkq603 PMC2938201 20601685

[39] Dobin, A. et al. STAR: Ultrafast universal RNA-seq aligner. *Bioinformatics* **29**, 15–21. 10.1093/bioinformatics/bts635 (2013). 23104886 10.1093/bioinformatics/bts635 PMC3530905

[40] McKenna, A. et al. The genome analysis toolkit: A MapReduce framework for analyzing next-generation DNA sequencing data. *Genome Res.* **20**, 1297–1303. 10.1101/gr.107524.110 (2010). 20644199 10.1101/gr.107524.110 PMC2928508

[41] Martin, M. Cutadapt removes adapter sequences from high-throughput sequencing reads. **17** 3 10.14806/ej.17.1.200 (2011).

[42] Langmead, B., Trapnell, C., Pop, M. & Salzberg, S. L. Ultrafast and memory-efficient alignment of short DNA sequences to the human genome. *Genome. Biol.* **10**, R25. 10.1186/gb-2009-10-3-r25 (2009). 19261174 10.1186/gb-2009-10-3-r25 PMC2690996

[43] Koboldt, D. C. et al. VarScan 2: Somatic mutation and copy number alteration discovery in cancer by exome sequencing. *Genome. Res.* **22**, 568–576. 10.1101/gr.129684.111 (2012). 22300766 10.1101/gr.129684.111 PMC3290792

[44] Koboldt, D. C., Larson, D. E. & Wilson, R. K. Using varscan 2 for germline variant calling and somatic mutation detection. *Curr. Protoc. Bioinform.*10.1002/0471250953.bi1504s44 (2013). 10.1002/0471250953.bi1504s44 PMC4278659 25553206

[45] Conticello, S. G. et al. Interaction between antibody-diversification enzyme AID and spliceosome-associated factor CTNNBL1. *Mol. Cell* **31**, 474–484. 10.1016/j.molcel.2008.07.009 (2008). 18722174 10.1016/j.molcel.2008.07.009

[46] Hill, J. T. et al. Poly peak parser: Method and software for identification of unknown indels using sanger sequencing of polymerase chain reaction products. *Dev. Dyn.* **243**, 1632–1636. 10.1002/dvdy.24183 (2014). 25160973 10.1002/dvdy.24183 PMC4525701

[47] Robinson, M. D., McCarthy, D. J. & Smyth, G. K. edgeR: A Bioconductor package for differential expression analysis of digital gene expression data. *Bioinformatics* **26**, 139–140. 10.1093/bioinformatics/btp616 (2010). 19910308 10.1093/bioinformatics/btp616 PMC2796818

[48] Xu, S. et al. Using clusterProfiler to characterize multiomics data. *Nat. Protoc.* **19**, 3292–3320. 10.1038/s41596-024-01020-z (2024). 39019974 10.1038/s41596-024-01020-z

