# Supplementary material for: C to U RNA editing of MFN1 is regulated by ADARB1 and associates with favourable prognosis in chronic lymphocytic leukemia
Source: Sci Rep. 2025 Aug 14;15:29856. doi: 10.1038/s41598-025-15666-6 (PMC12354836; doi:10.1038/s41598-025-15666-6)
Supplement: Supplementary file 1 — Supplementary Information 1. [file 41598_2025_15666_MOESM1_ESM.pdf]

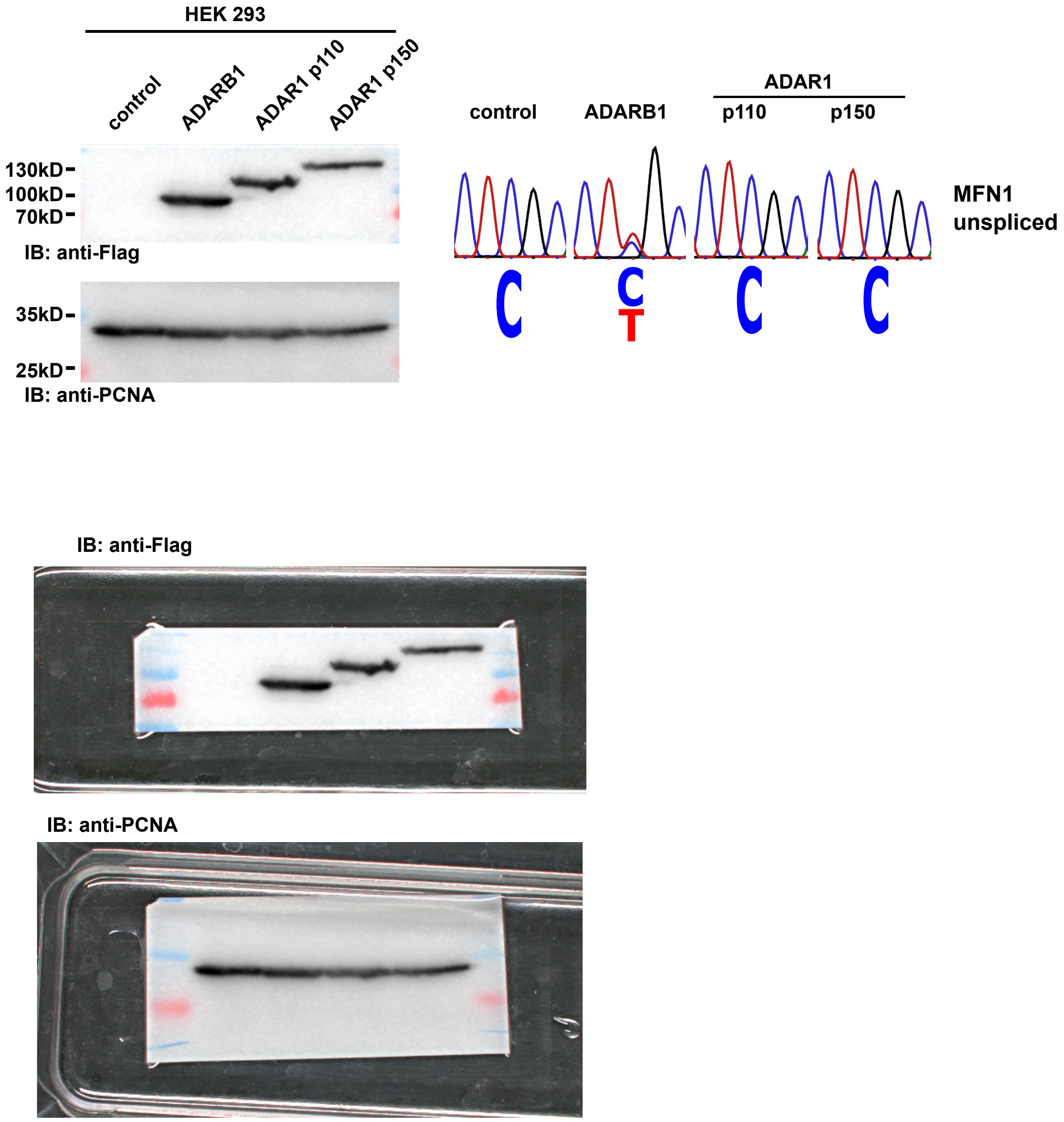


Supplementary Figure S1. Western blot analysis of HEK293 cells transfected with the respective Flag-tagged constructs (ADARB1, ADAR p110 and ADAR p150). Sanger sequencing of MFN1 C-to-U editing sites from unspliced MFN1 transcripts in HEK293 cells transfected with the respective Flag-tagged constructs is shown on the right. Original western blot membranes are shown below.

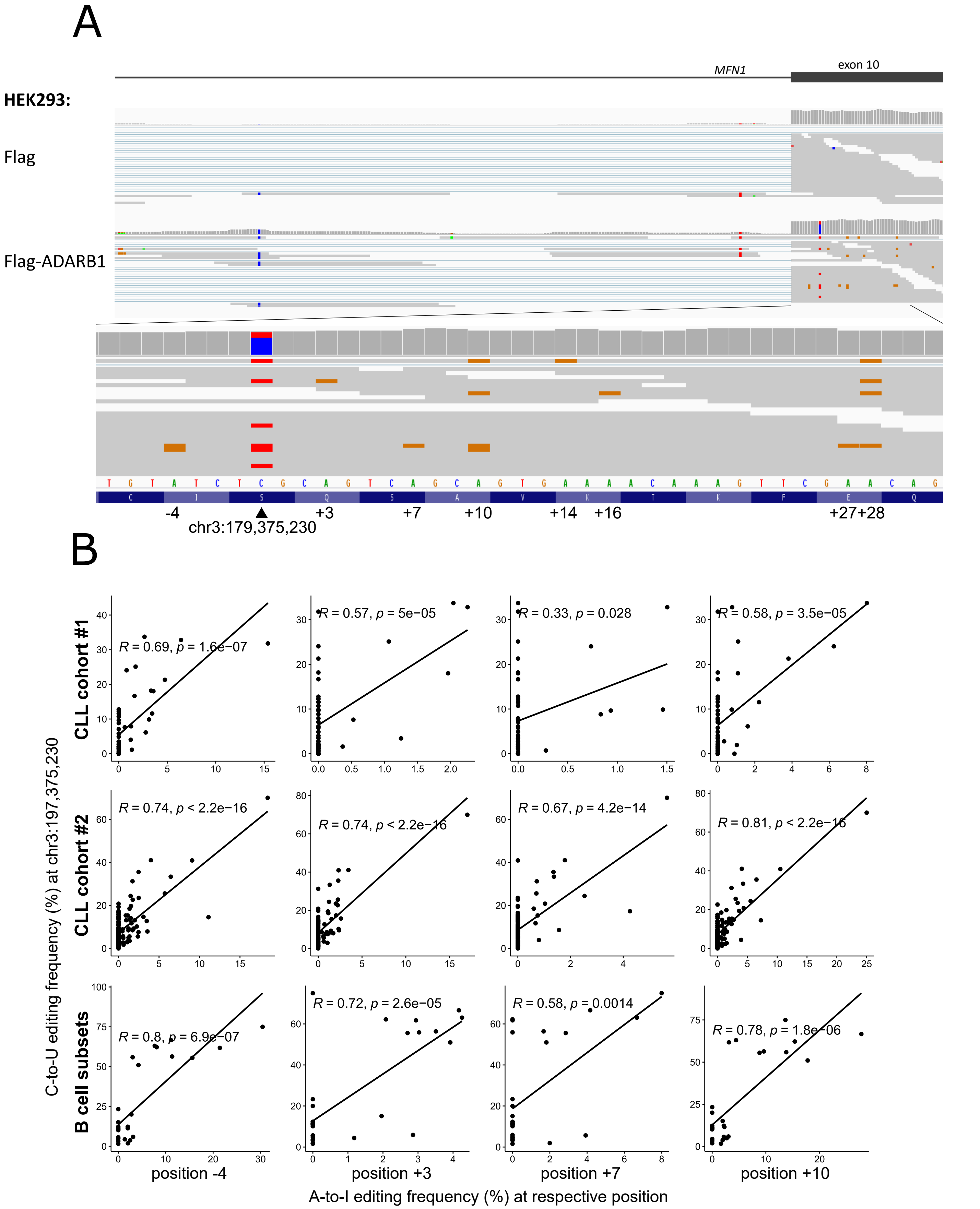


Supplementary Figure S2. A) A-to-I editing cluster at the C-to-U editing site in MFN1 identified in ADARB1 transfected versus control transfected HEK293 cell lines. B) Editing frequencies at position -4, +3, +7, +10 relative to C-to-U editing site are correlated with C-to-U editing frequencies in MFN1 transcripts in the two CLL cohorts and normal B cells.

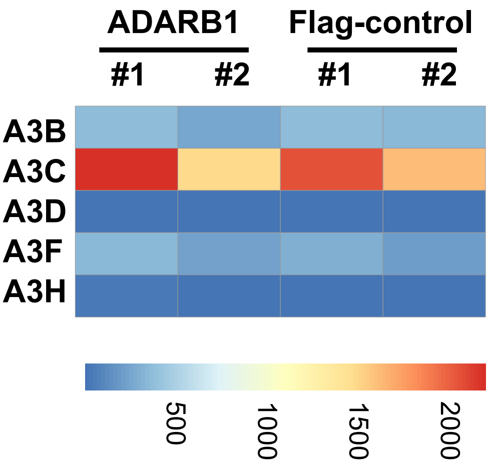


Supplementary Figure S3. Transcript read abundance of detectable AID/APOBEC3 members in HEK293 cells

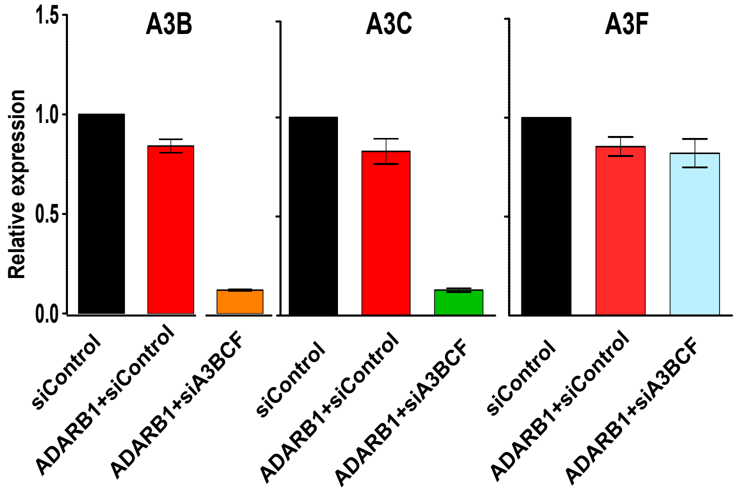


Supplementary Figure S4. Validation of siRNA knockdown of APOBEC3B, C, F in 293HEK cells.

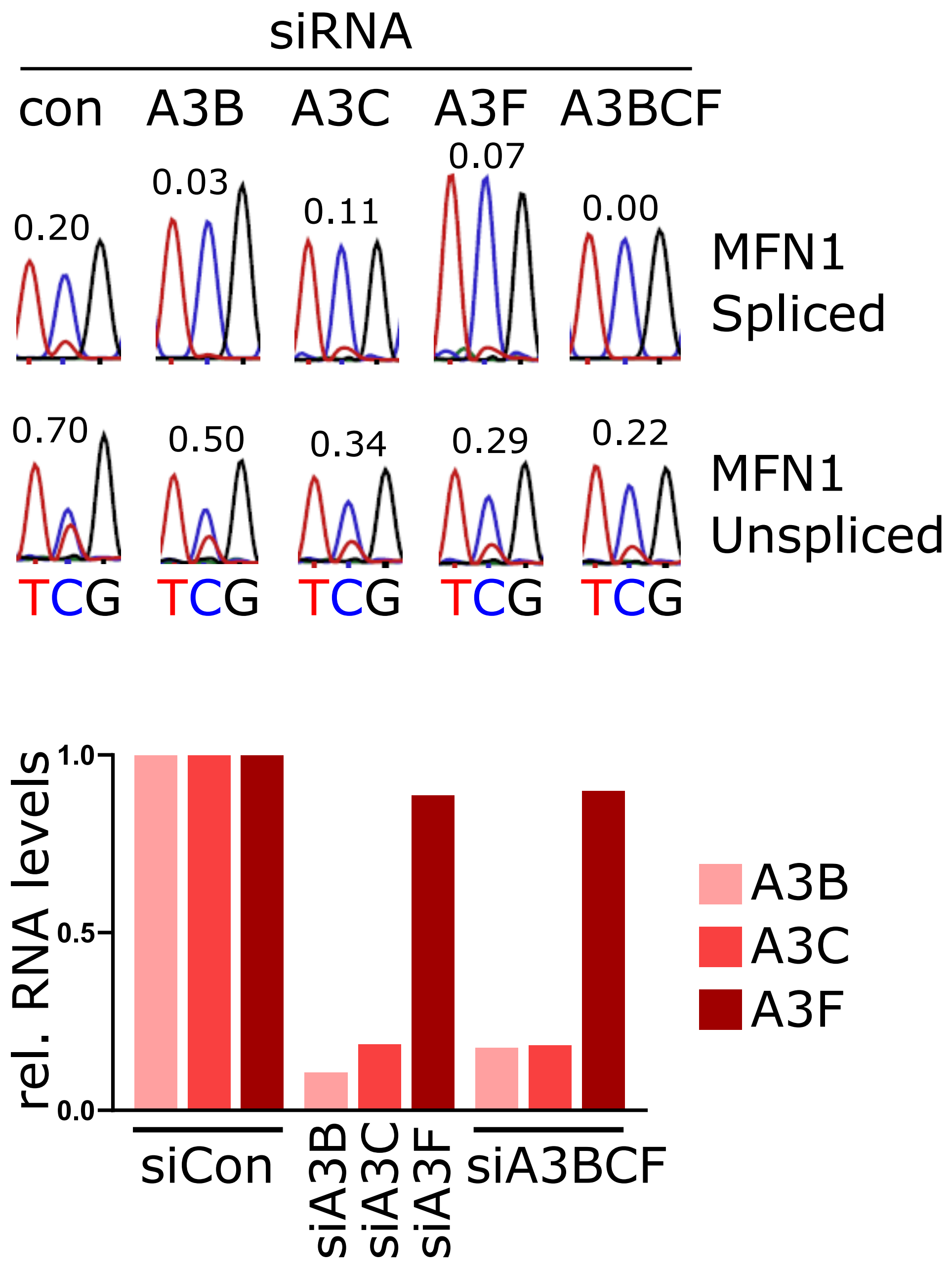


Supplementary Figure S5. Knockdown of individual APOBEC3B, C or F and pooled BCF in ADARB1 transfected HEK293 cells and effect on MFN1 C-to-U editing. Editing frequencies are indicated above the edited C. RNA levels of respective A3 members upon individual or pooled knockdowns are depicted below.

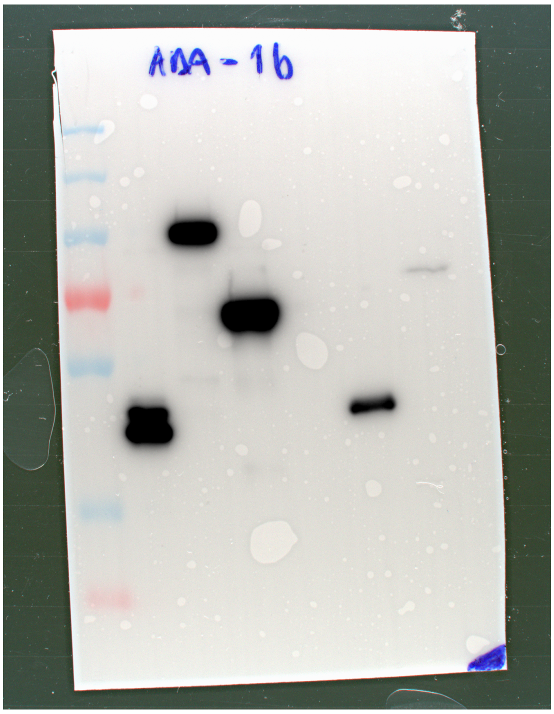


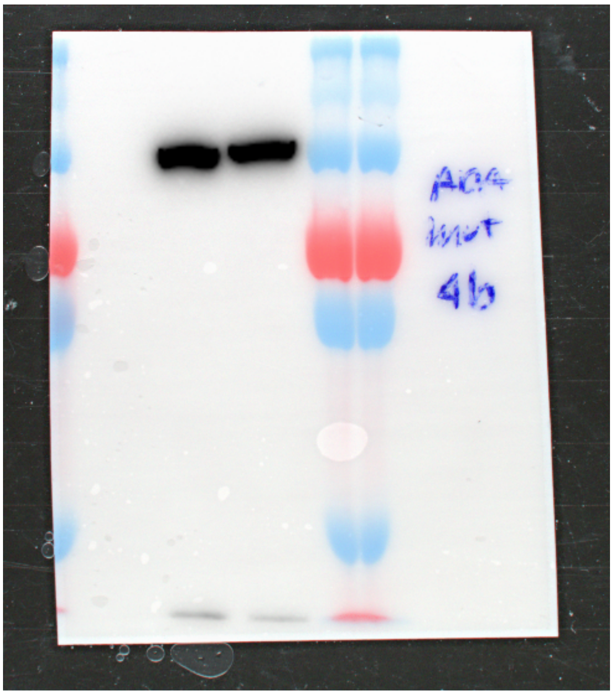


Supplementary Figure S6. Full-length blots from Fig 4E

# Overall Survivall

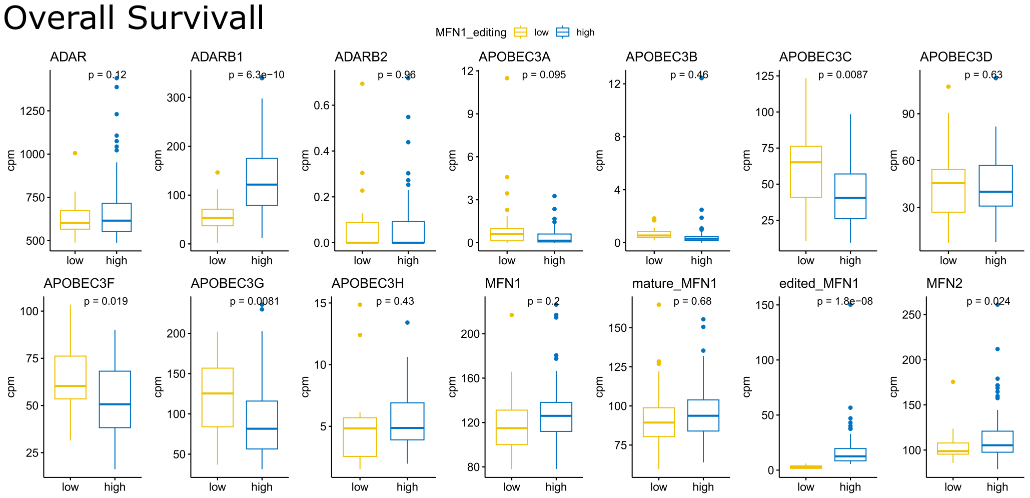


# TTT from diagnosis

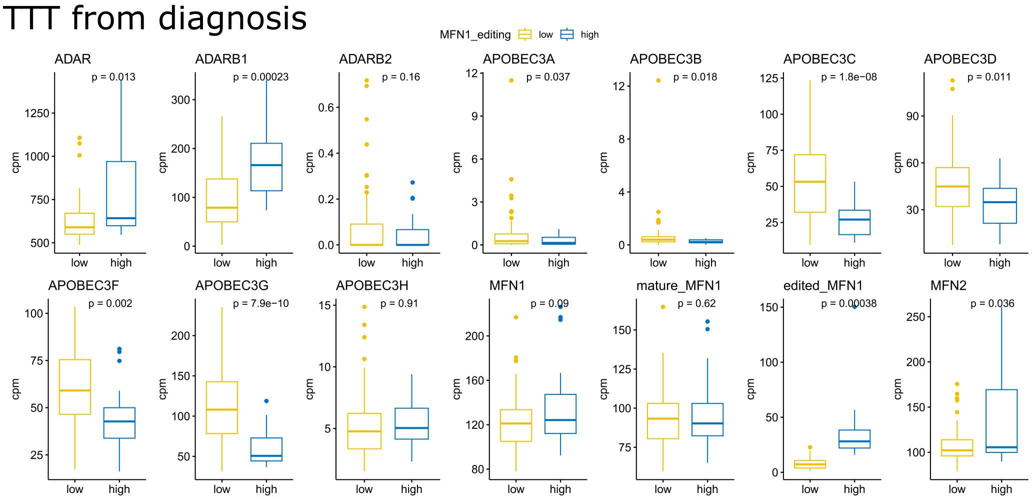


# TTT from sampling

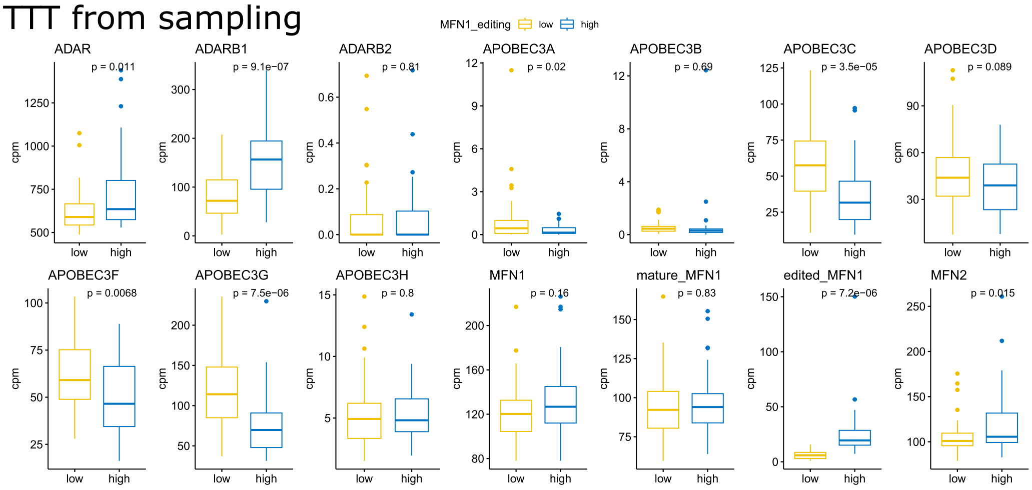


Supplementary Figure S7. Expression levels of ADARs APOBECs and MFN variants in CLL samples from the validation cohort from Fig.6B.
